# Distinct Hippocampal Expression Profiles of Long Non-coding RNAs in an Alzheimer’s Disease Model

**DOI:** 10.1007/s12035-016-0038-5

**Published:** 2016-08-08

**Authors:** Bo Yang, Zi-an Xia, Bingwu Zhong, Xingui Xiong, Chenxia Sheng, Yang Wang, Wei Gong, Yucheng Cao, Zhe Wang, Weijun Peng

**Affiliations:** 1 Department of Integrated Traditional Chinese and Western Medicine, Xiangya Hospital, Central South University, Changsha, Hunan 410008 China; 2 Department of Integrated Traditional Chinese & Western Medicine, The Second Xiangya Hospital, Central South University, Changsha, Hunan 410011 China

**Keywords:** Alzheimer’s disease, Long non-coding RNAs (lncRNAs), Microarray, Hippocampus, Expression profiles

## Abstract

Alzheimer’s disease (AD), the most prevalent form of dementia worldwide, is a complex neurodegenerative disease characterized by the progressive loss of memory and other cognitive functions. The pathogenesis of AD is not yet completely understood. Although long non-coding RNAs (lncRNAs) have recently been shown to play a role in AD pathogenesis, the specific influences of lncRNAs in AD remain largely unknown; in particular, hippocampal lncRNA expression profiles in AD rats are lacking. In this study, microarray analysis was performed to investigate the hippocampal expression patterns of dysregulated lncRNAs in a rat model of AD. A total of 315 lncRNAs and 311 mRNAs were found to be significantly dysregulated in the AD model (≥2.0 fold, *p* < 0.05). Then, quantitative real-time PCR was used to validate the expression of selected lncRNAs and mRNAs. Bioinformatics tools and databases were employed to explore the potential lncRNA functions. This is the first study to comprehensively identify dysregulated hippocampal lncRNAs in AD and to demonstrate the involvement of different lncRNA expression patterns in the hippocampal pathogenesis of AD. This information will enable further research on the pathogenesis of AD and facilitate the development of novel AD therapeutics targeting lncRNAs.

## Introduction

Alzheimer’s disease (AD) is considered to be the most common cause of dementia; AD is a progressive neurodegenerative disease characterized by the accumulation of amyloid-β(Aβ) plaques and neurofibrillary tangles, synaptic and neuronal loss, and cognitive decline [1]. In the USA alone, an estimated 5.2 million individuals aged 65 and older have AD, and this number is expected increase to 13.8 million by 2050 [2]. Unfortunately, no currently available therapeutic strategies for AD slow or stop the neuronal damage that causes AD symptoms and eventually results in death [3, 4]. Thus, there is an urgent need for novel strategies of improving our mechanistic understanding of AD, which could lead to the discovery of novel therapeutic targets.

Long non-coding RNAs (lncRNAs), a subclass of ncRNAs, are most commonly defined as the transcripts of more than 200 nucleotides that structurally resemble mRNAs but have no protein-coding capacity [5]. With the development of techniques to detect lncRNA, accumulating evidence indicates that lncRNAs participate in a wide variety of important biological phenomena, such as imprinting genomic loci, influencing chromosome conformation and allosterically regulating enzymatic activity [6]. Moreover, multiple lines of evidence have linked lncRNA mutations and dysregulation with diverse human diseases, ranging from different types of cancer and neurodegeneration to gynaecological diseases [7–9].

The role of lncRNAs in AD has attracted considerable attention. Recent studies have further confirmed the involvement of certain lncRNAs in AD [10–13]. In addition, with recent advancements in transcriptome-wide profiling, numerous AD-associated lncRNAs have been discovered. Furthermore, the dysregulation of lncRNA expression in post-mortem tissue samples from AD patients [14, 15] and transgenic AD animals [16] has been investigated. Despite these findings, the expression patterns, targets, and functions of lncRNAs involved in the pathogenesis of AD remain largely unknown [17]. Therefore, further research is of great importance.

In addition, as a crucial component of the medial temporal lobe memory circuit, the hippocampus is affected early in AD and displays synaptic and intraneuronal molecular remodelling against a pathological background of extracellular Aβ deposition and intracellular neurofibrillary tangle formation in the early stages of AD [18]. Moreover, behavioural studies have long suggested that the hippocampus plays a critical role in learning and memory, which depend on functional and structural changes occurring in the hippocampus, such as long-term potentiation and synaptic remodelling [19].

Therefore, in the present study, we applied microarray technology to analyse the expression profiles of lncRNAs and messenger RNAs (mRNAs) in the hippocampus of rats in a validated AD model. Additionally, gene ontology (GO) and Kyoto Encyclopedia of Genes and Genomes (KEGG) analyses were performed to predict the biological roles and potential signalling pathways of these differentially expressed lncRNAs. Moreover, an lncRNA-mRNA network analysis was conducted to further explore the potential roles of differentially expressed lncRNAs in AD pathogenesis.

## Materials and Methods

### Ethics Statement

All animal protocols were approved by the Central South University (Changsha, China) and were performed in compliance with the National Institutes of Health Guide for the Care and Use of Laboratory Animals. This investigation was conducted in accordance with ethical standards and the Declaration of Helsinki, as well as according to national and international guidelines. This research was approved by the authors’ institutional review board.

### Animals and Experimental Design

A total of 20 adult, male Sprague–Dawley rats (250 ± 30 g) were purchased from the Laboratory Animal Centre of Central South University. The animals were housed under controlled conditions (12-h light/dark cycle, 25 °C, 50 ± 10 % relative humidity) with water and food pellets available ad libitum.

The animals were randomly divided into the following two groups (*n* = 10 in each group): (1) the AD group and (2) the control group.

### Surgery

In this experiment, we utilized an intracerebroventricular (ICV) injection of Aβ1–42 oligomers into the cerebral ventricles of the animals to induce a validated AD model, as we have previously described [20]. In brief, the animals were anaesthetised with 10 % chloral hydrate (4 ml/kg) and placed in a stereotactic frame. The Aβ_1–42_ oligomers(5 μl*2, Sigma, St. Louis, MO, USA) were injected bilaterally into the lateral ventricles through a stainless steel cannula by using the following coordinates: 1.1 mm posterior to the bregma, 2.2 mm lateral to the sagittal suture, and 3.0 mm beneath the dura. For the control group, the vehicle was injected bilaterally into the lateral ventricles through a stainless steel cannula by using the above-mentioned coordinates.

### Sample Collection

After the Morris water maze test was completed, all rats were anaesthetised with chloral hydrate and were sacrificed via decapitation. Their hippocampal tissues were stored in liquid nitrogen followed by storage at −80 °C prior to analysis.

### Morris Water Maze Test

Spatial learning and memory deficits were evaluated using the Morris water maze test as we have previously described [21], with additional modifications. In brief, the test was conducted at 31–35 days after injury. Initial training was conducted on days 31–34 after injury; during this period, we trained the rats to locate a hidden, submerged platform using peripheral visual information. The rats were introduced into a pool at one of four entry points, and every entry point was used over the course of a day. The rats were given 60 s to locate the platform and were allowed to remain on the platform for 10 s before being removed. Rats that were unable to locate the platform within 60 s were placed on the platform for 10 s before being removed from the pool. On the 35th day, a probe trial was conducted, in which the platform was removed and the number of crossings over the previous platform location was recorded over one 60-s trial. An overhead video camera connected to the ANY-maze video tracking system (Stoelting Co., USA) was used to record all the trials and track the movements of the animals.

### RNA Extraction

Total RNA was extracted from each hippocampal tissue sample by soaking the tissue samples in TRIzol Reagent (Invitrogen, Grand Island, NY, USA) in accordance with the manufacturer’s instructions. RNA quantity and quality were measured using a Nano Drop ND-1000, and RNA integrity was assessed by standard denaturing agarose gel electrophoresis.

### Microarray Analysis

Sample labelling and array hybridization were performed according to the Agilent One-Colour Microarray-Based Gene Expression Analysis protocol (Agilent Technology), with minor modifications. In brief, mRNA was purified from total RNA after the removal of rRNA (mRNA-ONLY™ Eukaryotic mRNA Isolation Kit, Epicentre). Then, each sample was amplified and transcribed into fluorescent cRNA along the entire length of the transcripts without 3′ bias via a random priming method (Arraystar Flash RNA LabellingKit, Arraystar). The labelled cRNAs were purified using an RNeasy Mini Kit (Qiagen). The concentration and specific activity of the labelled cRNAs (pmol Cy3/μg cRNA) were measured using the NanoDrop ND-1000. In all, 1 μg of each labelled cRNA was fragmented by adding 5 μl of 10 × Blocking Agent and 1 μl of 25 × Fragmentation Buffer and heating the mixture at 60 °C for 30 min. Then, 25 μl of 2 × GE Hybridization Buffer was added to dilute the labelled cRNA. Subsequently, 50 μl of hybridization solution was dispensed into the gasket slide, which was then assembled with the lncRNA expression microarray slide. The slides were incubated for 17 h at 65 °C in an Agilent Hybridization Oven. The hybridized arrays were washed, fixed and scanned using an Agilent DNA Microarray Scanner (part number G2505C). Microarray hybridization and expression data collection were performed by KangChen Bio-tech, Shanghai, China.

### Quantitative Real-Time-PCR Validation

As previously described [22], total RNA was reverse-transcribed into cDNA using SuperScript III Reverse Transcriptase(Invitrogen, Grand Island, NY, USA) in accordance with the manufacturer’s instructions. An Applied Biosystems ViiA 7 Real-Time PCR System and 2 × PCR Master Mix were used to perform quantitative real-time (qRT)-PCR (Arraystar). The reaction conditions were as follows: incubation at 95 °C for 10 min, followed by 40 cycles of 95 °C for 10 s and 60 °C for 1 min. The relative lncRNA and mRNA expression levels were calculated using the 2^−ΔΔCt^ method and were normalized to GAPDH, as an endogenous reference transcript [23]. The specific primers for each gene are listed in Table 1. The data represent the means of three experiments.

**Table 1:** Primers designed for qRT-PCR validation of candidate lncRNAs and mRNAs

	Forward primer	Reverse primer	Product length	Tm(°C)
BC092582	5′ -GGAGGTGAATGCTGAGGAGGA-3′	5′ - ATGAAGGTAGAGGCGGTGGTC-3′	60	60
MRAK050857	5′ -CCCCCAAGAACGGTGGAGTG-3′	5′ -GGAGACAGCGCCTGAGAACGAG3′	120	60
MRAK088596	5′ -AGGGGTAACGAACAACAAAGA-3′	5′ - CATGGTACTCAGAATGCTAAAAT-3′	59	60
MRAK081790	5′ -AAAATTGGTTGAGCTGGTATAGGT-3′	5′ -CCTTGGCATCAGTTTCCTTGT-3′	168	60
MAPK10	5′-ATGTTAGTGATTGACCCAGCGAAG-3′	5′-TGCTCCCTTTCATCCAGTTGC-3′	60	63
S100a8	5′ -GGGAATCACCATGCCCTCTAC-3′	5′ -GCCCACCCTTATCACCAACAC-3′	60	168
GAPDH	5′ -GCTCTCTGCTCCTCCCTGTTCTA-3′	5′-TGGTAACCAGGCGTCCGATA-3′	60	124

### GO Annotations and KEGG Pathways

GO annotations and pathway analysis were applied to investigate the roles of all differentially expressed mRNAs, as previously described [24, 25]. In brief, GO analysis was applied to elucidate genetic regulatory networks of interest by forming hierarchical categories according to the molecular function, biological process, and cellular component aspects of the differentially expressed genes (http://www.geneontology.org). Pathway analysis was performed to explore the significant pathways of the differentially expressed genes, according to KEGG (http://www.genome.jp/kegg/).

### Construction of Co-Expression Network

To identify interactions among the differentially expressed lncRNAs and mRNAs, we constructed a co-expression network based on a correlation analysis of the differentially expressed lncRNAs and mRNAs [26]. The network was constructed according to the normalized signal intensities of specific mRNA and lncRNA expression levels. Pearson’s correlation coefficients equal to or greater than 0.7 were used to identify the lncRNAs and coding genes. Then, the lncRNA-mRNA co-expression network was constructed using Cytoscape software (The Cytoscape Consortium, San Diego, CA, USA).

### Statistical Analysis

All data are shown as the mean ± standard error of the mean (SEM) and were analysed using the statistical software SPSS version 22.0 (SPSS Inc., Chicago, IL, USA). Student’s *t* test was performed for comparisons between two groups, whereas ANOVA was performed for repeated measures. The false discovery rate was calculated to correct the *p* value. Differences with *p* < 0.05 were considered statistically significant. Fold change (FC) and Student’s *t* test were used to analyse the statistical significance of the microarray results. FC ≥ 2 and *p* < 0.05 were considered the threshold values for designating differentially expressed lncRNAs and mRNAs.

## Results

### Morris Water Maze Test

**Fig. 1:**
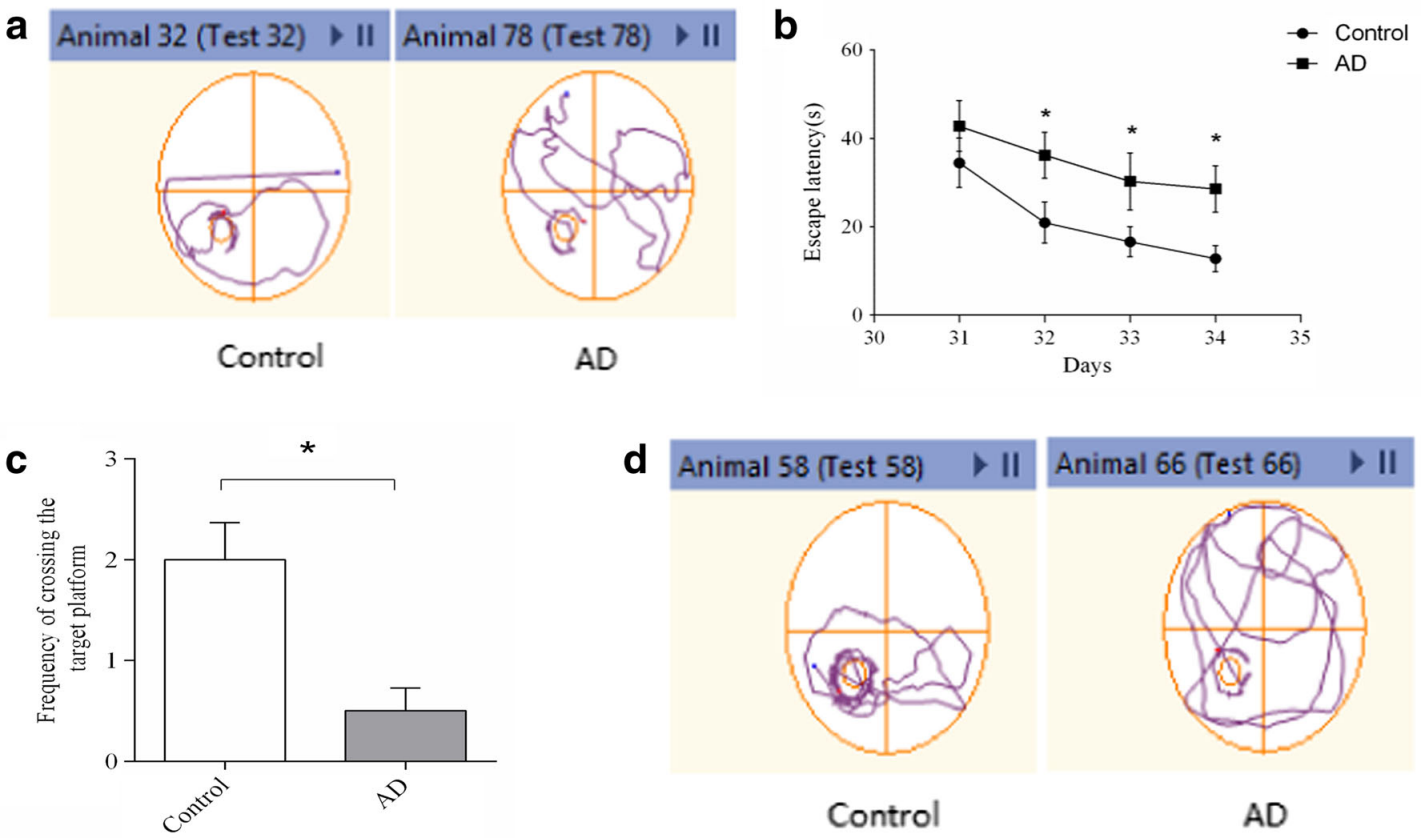
Spatial learning and memory was assessed in Morris water maze test. **a** Representative images of the swim paths on day 34, showing that rats in the control group were able to find the hidden platform more easily than the AD rats and indicating that the rats in the AD group (31 s) required a longer time to find the hidden platform than the rats in the control group (15 s); these results suggested that Aβ_1–42_ impaired spatial learning ability. **b** Escape latency. A significant difference was detected between the control and AD groups on days 32–34 (*p < 0.05). **c** Probe test. The frequency of crossing the target platform was recorded as an indicator of spatial memory. Significant differences were observed between the control and AD groups (*p < 0.05). **d** Representative images of the frequency of crossing the target platform, showing that rats in the control group had a crossing frequency of approximately 3 times, while that of rats in the AD group was reduced to approximately 1 time, suggesting that there was significantly better performance in the control group compared with the AD group; *n* = 6 rats/group. Data are expressed as the mean ± standard error of the mean (SEM)

Spatial learning was evaluated on days 31–34 after the rats received an ICV Aβ_1–42_ injection, and a probe trial for spatial memory was conducted on day 35. As expected, rats injected with Aβ_1–42_ were less able than mice in the control group to find the platform and learn its location by the 34th day of training, indicating poor learning performance (Fig. 1b). In addition, compared with animals in the control group, the Aβ_1–42_-treated animals performed poorly and failed to recollect the location of the submerged platform on the 35th day, demonstrated by significantly fewer crossings over the previous platform location (Fig. 1c).

### lncRNA and mRNA Expression Profiles in AD

**Fig. 2:**
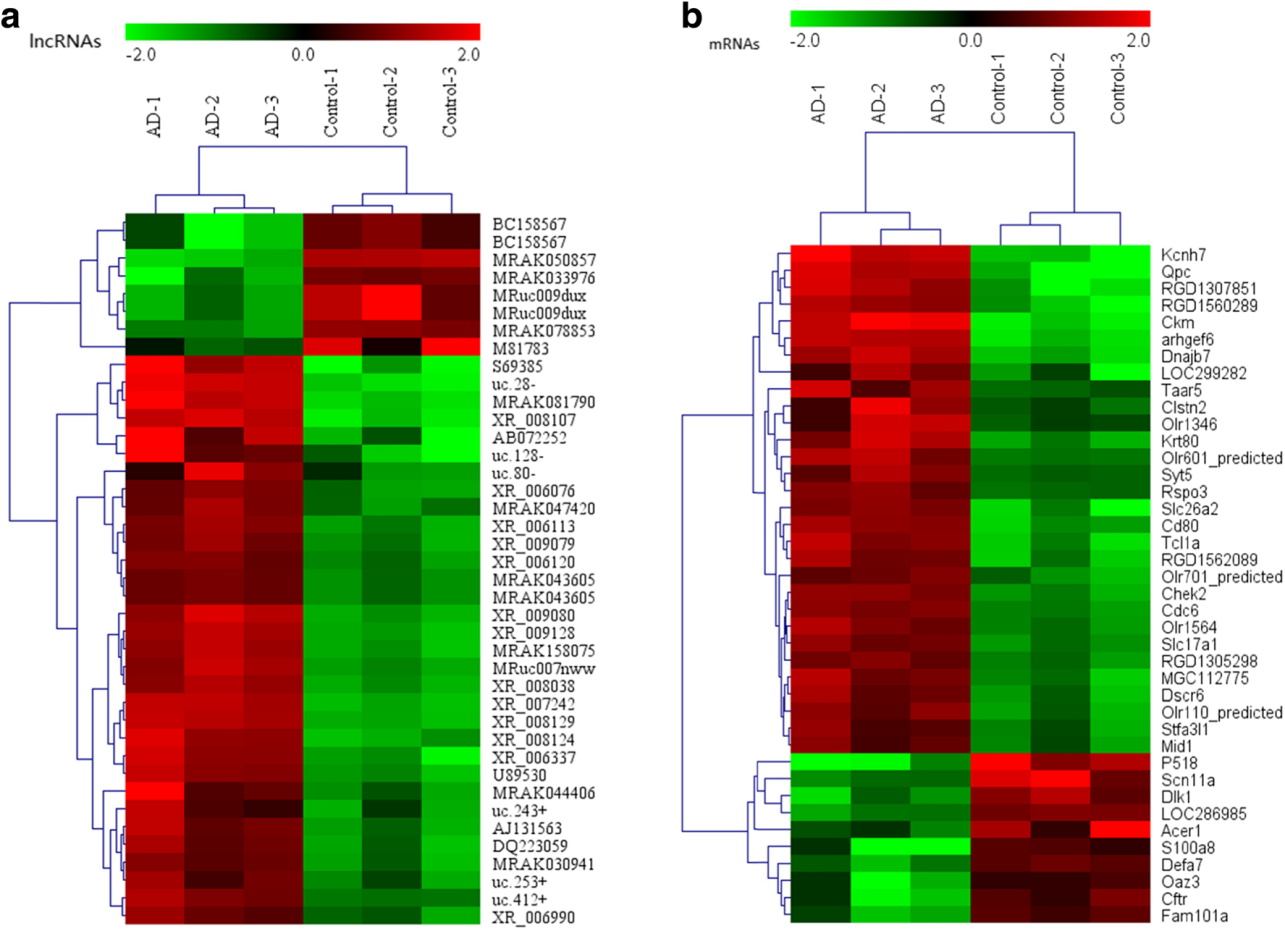
The heat map and hierarchical clustering of the top 40 differentially expressed lncRNAs (**a**) and mRNAs (**b**) between AD and control hippocampal samples. The data are depicted as a data matrix, in which each row represents one lncRNA (mRNA) and each column represents one of the hippocampal samples. The relative lncRNA (mRNA) expression is depicted according to the colour scale shown at the top. *Red* represents high relative expression, and *green* represents low relative expression; −2.0, 0 and 2.0 are FCs in the corresponding spectrum. The magnitude of deviation from the median is represented by the colour saturation

**Fig. 3:**
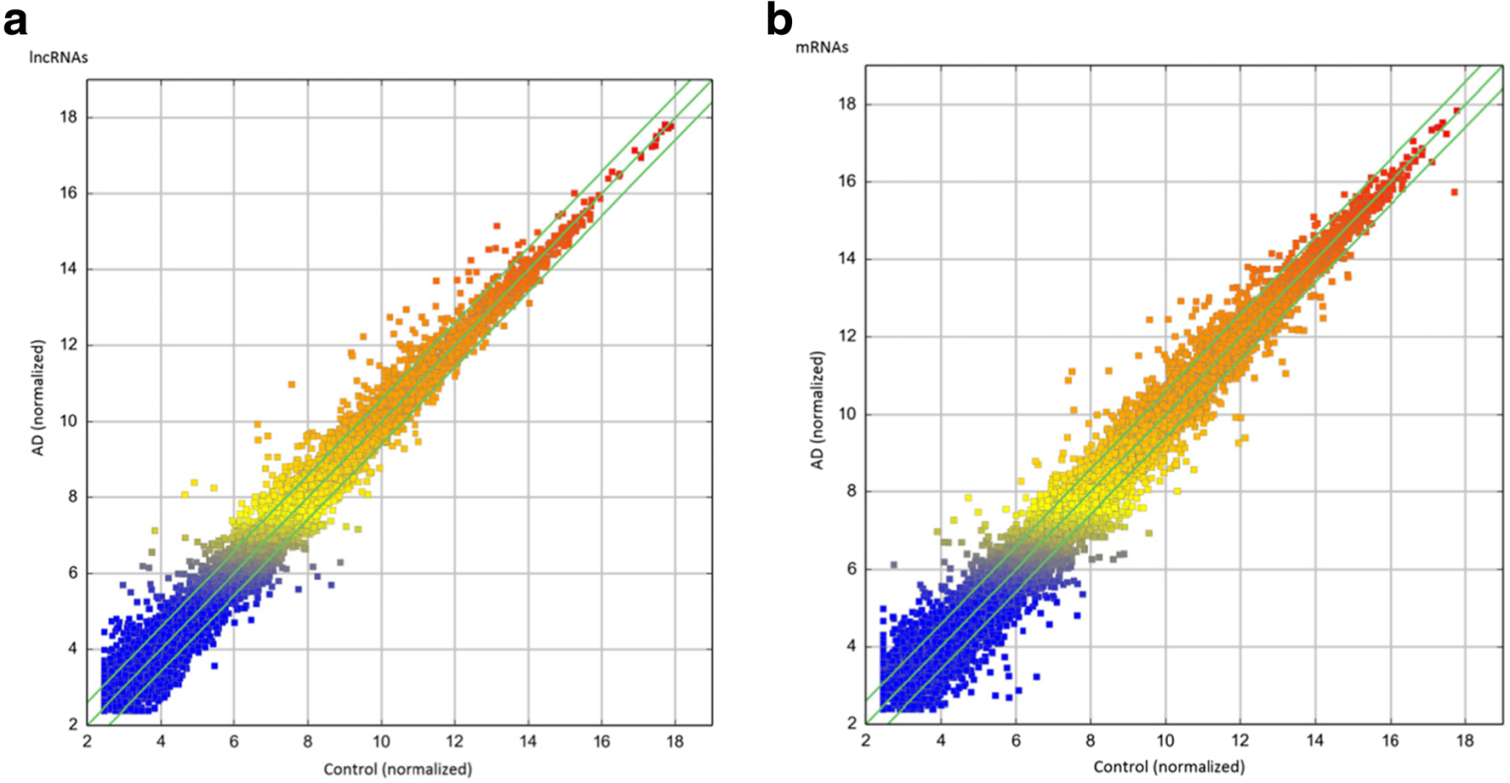
The scatter plot of lncRNA (**a**) and mRNA (**b**) expression variation between the AD and control hippocampal samples. The values shown on the x-axis and y-axis in the scatter plot are the normalized signal values of each sample (log2 scale). The *green lines* are fold-change lines (the default fold-change value given is 2.0). The lncRNAs (mRNAs) *above the top green line and below the bottom green line* showed an FC >2.0 in expression between the 2 compared samples

Microarray analysis was used to assess the expression levels of lncRNAs in AD rats relative to those in control rats. We identified 315 significantly dysregulated lncRNAs in the AD rats: 238 were up-regulated, while 77 were down-regulated (≥2.0 fold, *p* < 0.05). The top 40 most significantly differentially expressed lncRNAs are shown in Table 2. Among the dysregulated lncRNA transcripts, S69385 was the most down-regulated, with an FC of 11.25, whereas MRAK050857 was the most up-regulated, with an FC of 7.65. The variation of lncRNA expression between the AD and control rats is shown with a scatter plot (Fig. 2a). The clustering analysis revealed the relationships among lncRNA expression patterns in different samples (Fig. 3a).

**Table 2:** Top 40 aberrantly expressed lncRNAs in microarray analysis

SeqID	*P* value	Fold change	Regulation	Chr	Strand	Relationship	AD-1	AD-2	AD-3	Control-1	Control-2	Control-3
BC158567	0.01001	4.58596	Down	20	+	sense_intron_overlap	8.00638	6.43733	7.03962	9.36119	9.59565	9.11816
BC158567	0.01001	4.58596	Down	20	+	sense_exon_overlap	8.00638	6.43733	7.03962	9.36119	9.59565	9.11816
MRAK050857	0.00001	7.65238	Down	10	−	others	5.53087	5.65205	5.90255	8.58667	8.61557	8.69096
MRAK033976	0.00288	5.04632	Down	X	−	others	5.29233	6.53179	5.94278	8.28533	8.20126	8.28601
MRuc009dux	0.00330	6.00053	Down	4	+	sense_intron_overlap	6.03729	6.69110	6.14928	8.93886	9.46715	8.22693
MRuc009dux	0.00330	6.00053	Down	4	+	antisense_intron_overlap	6.03729	6.69110	6.14928	8.93886	9.46715	8.22693
MRAK078853	0.00005	4.46748	Down	4	+	sense_exon_overlap	5.67166	5.69835	5.37772	7.81646	7.78834	7.62132
M81783	0.04499	3.88207	Down	6	−	sense_exon_overlap	6.54969	5.91603	6.06684	8.45648	6.87919	9.06737
S69385	0.00244	11.25543	Up	8	+	sense_exon_overlap	9.22457	7.80020	8.15135	4.55781	5.46104	4.67963
uc.28-	0.00002	10.74721	Up	2	+	antisense_intron_overlap	8.27963	8.00554	7.92272	4.83231	4.62756	4.47034
MRAK081790	0.00022	10.61600	Up	X	−	sense_intron_overlap	11.47399	10.67374	10.76289	7.51004	7.73699	7.43908
XR_008107	0.00003	9.80265	Up	X	+	others	9.89685	10.08962	9.80230	6.48063	6.90673	6.52189
AB072252	0.02153	9.80128	Up	1	−	others	8.06770	6.20245	7.09967	4.11682	4.90890	2.46520
uc.128-	0.01416	6.99610	Up	8	+	intergenic	7.46133	6.05214	6.17050	4.63293	3.73602	2.89537
uc.80-	0.02034	4.03223	Up	3	−	sense_intron_overlap	4.03512	5.57163	4.80373	3.40618	2.50436	2.46520
XR_006076	0.00058	4.22020	Up	18	−	others	4.55077	4.92157	4.75323	3.02408	2.50436	2.46520
MRAK047420	0.00084	4.01561	Up	5	+	sense_intron_overlap	5.46621	6.04554	5.64582	3.90005	3.43310	3.80757
XR_006113	0.00019	4.89262	Up	3	+	sense_intron_overlap	12.79504	13.15587	12.89520	10.60371	10.91719	10.45340
XR_009079	0.00043	4.43645	Up	7	+	others	8.96824	9.31937	8.92314	6.92116	7.19221	6.64916
XR_006120	0.00028	3.95666	Up	3	+	intergenic	12.37788	12.39927	12.13337	10.29845	10.57616	10.08305
MRAK043605	0.00012	3.71597	Up	10	+	antisense_exon_overlap	9.04173	9.15901	8.99951	7.05250	7.40744	7.05909
MRAK043605	0.00012	3.71597	Up	10	+	antisense_exon_overlap	9.04173	9.15901	8.99951	7.05250	7.40744	7.05909
XR_009080	0.00018	6.67415	Up	1	−	others	11.91682	12.54961	12.23871	9.43643	9.68088	9.37209
XR_009128	0.00004	6.54726	Up	16	−	intergenic	9.47314	9.82283	9.57302	6.93783	7.06204	6.73645
MRAK158075	0.00013	6.21123	Up	19	+	antisense_exon_overlap	11.63517	12.06746	11.72937	9.16909	9.39121	8.96705
MRuc007nww	0.00018	5.77962	Up	6	+	intergenic	12.46794	13.04085	12.73809	10.17715	10.38814	10.08866
XR_008038	0.00008	5.72687	Up	2	−	intergenic	11.56619	11.93042	11.66727	9.13306	9.41106	9.06652
XR_007242	0.00001	7.29233	Up	15	+	intergenic	9.60316	9.58071	9.36365	6.59651	6.79242	6.55945
XR_008129	0.00001	7.07133	Up	16	−	intergenic	8.37982	8.26123	8.10604	5.44713	5.51678	5.31723
XR_008124	0.00034	6.46782	Up	18	−	others	6.60833	6.00739	5.97723	3.32404	3.46613	3.72295
XR_006337	0.00099	6.59840	Up	12	−	intergenic	6.06002	5.51444	5.50684	3.15227	3.29748	2.46520
U89530	0.00040	5.56718	Up	3	−	intergenic	6.49347	6.00548	5.95748	3.73959	3.89338	3.39263
MRAK044406	0.01616	4.85928	Up	2	−	sense_exon_overlap	7.99810	6.33157	6.47274	4.56294	5.03938	4.35786
uc.243+	0.01513	3.76335	Up	2	−	intergenic	9.44347	8.51668	8.32754	6.51866	7.48587	6.54710
AJ131563	0.00182	4.62465	Up	16	+	sense_exon_overlap	14.15639	13.39135	13.57111	11.38921	11.87005	11.23156
DQ223059	0.00212	4.47442	Up	10	+	others	12.99165	12.36331	12.52671	10.34195	10.93173	10.12288
MRAK030941	0.00135	4.04066	Up	4	+	sense_exon_overlap	15.33834	14.99113	15.12357	13.02945	13.58079	12.79903
uc.253+	0.00427	3.70040	Up	5	+	intergenic	11.93550	11.18617	11.56148	9.58592	10.12549	9.30870
uc.412+	0.00022	3.98847	Up	10	−	sense_intron_overlap	4.76540	4.37265	4.24680	2.42778	2.50436	2.46520
XR_006990	0.00213	3.59883	Up	Un	+	intergenic	5.02968	4.64237	4.48417	3.01448	3.13395	2.46520

SeqID: lncRNA name. *P* value:P value calculated from unpaired t-test. Fold Change: the absolute ratio (no log scale) of normalized intensities between two groups(AD vs Control). Chr: chromosome no. which lncRNA is transcribed. Strand: the strand of chromosome which lncRNA is transcribed; ‘+’ is sense strand of chromosome, ‘−’ is antisense strand of chromosome. Relationship: “sense exon overlap”: the LncRNA’s exon is overlapping a coding transcript exon on the same genomic strand; “sense intron overlap”: the LncRNA is overlapping the intron of a coding transcript on the same genomic strand; “antisense_exon_overlap”: the LncRNA is transcribed from the antisense strand and overlapping with a coding transcript; “antisense_intron_overlap”: the LncRNA is transcribed from the antisense strand without sharing overlapping exons;"bidirection”: the LncRNA is oriented head to head to a coding transcript within 1000 bp; “intergenic”: there are no coding transcripts within 30 kb of the LncRNA; “others”: means other LncRNAs. AD1–3,Control1–3:Normalized Intensity of each sample (log2 transformed)

Using microarray analysis, we identified 311 significantly dysregulated mRNAs in the AD rats: 191 were up-regulated, while 120 were down-regulated (≥2.0 fold, *p* < 0.05). The top 40 most significantly differentially expressed mRNAs are shown in Table 3. The most up-regulated and down-regulated mRNA transcripts were Ckm(NM_012530) and P518 (NM_198200), with an FC of 12.08 and 10.05, respectively. The variation of mRNA expression between the AD and control rats is shown with a scatter plot (Fig. 2b). The clustering analysis revealed the relationships among mRNA expression patterns in different samples (Fig. 3b).

**Table 3:** Top 40 aberrantly expressed mRNAs in microarray analysis

SeqID	GeneSymbol	*P* value	Fold change	Regulation	Chr	AD-1	AD-2	AD-3	Control-1	Control − 2	Control − 3
NM_131912	Kcnh7	0.00037	11.06140	Up	3	11.30127	10.61836	10.70082	7.60876	7.65469	6.95462
NM_001025134	Qpc	0.00032	10.39597	Up	10	6.37899	5.97278	6.02696	3.27530	2.50436	2.46520
NM_001013861	RGD1307851	0.00069	8.45568	Up	10	7.27314	6.97233	6.68490	4.41475	3.46865	3.80720
NM_001134623	RGD1560289	0.00132	7.65156	Up	4	7.46718	7.21876	7.13580	4.89982	4.42654	3.68811
NM_012530	Ckm	0.00006	12.08045	Up	1	10.79360	11.35903	11.15888	7.39529	7.74974	7.38268
NM_001005565	arhgef6	0.00002	8.69988	Up	X	7.92966	7.82064	7.80281	4.57567	4.98291	4.63155
NM_001130510	Dnajb7	0.00009	7.37606	Up	7	12.31645	12.68693	12.35062	9.52755	9.82563	9.35227
NM_182474	LOC299282	0.03959	6.15219	Up	6	6.26476	7.15561	6.68019	4.53369	5.23837	2.46520
NM_001009650	Taar5	0.00323	3.85177	Up	1	7.59855	6.56191	7.18826	5.07491	5.14253	5.29471
NM_134377	Clstn2	0.01116	3.67028	Up	8	10.99065	12.38604	11.62870	9.79077	10.00038	9.58658
NM_001000520	Olr1346	0.00853	3.56797	Up	9	6.82078	7.98940	7.87742	5.57183	5.83954	5.77092
NM_001008815	Krt80	0.00069	5.82118	Up	7	12.52671	13.29509	12.98709	10.29545	10.68643	10.20308
NM_001000512	Olr601_predicted	0.00034	4.60897	Up	3	4.79276	4.92615	4.29177	2.42778	2.50436	2.46520
NM_019350	Syt5	0.00086	3.58122	Up	1	12.20464	12.90256	12.50704	10.63153	10.75588	10.70548
NM_001100990	Rspo3	0.00012	3.52906	Up	1	9.81657	9.94154	9.54918	7.87261	7.97046	8.00638
NM_057127	Slc26a2	0.00126	5.92500	Up	18	10.07056	10.23371	10.05481	7.40892	8.14971	7.09999
NM_012926	Cd80	0.00021	5.62333	Up	11	7.63253	7.42776	7.38456	4.64968	5.24624	5.07467
NM_001109601	Tcl1a	0.00082	6.24817	Up	6	11.46388	10.90778	11.00265	8.32631	8.94392	8.17378
NM_001130502	RGD1562089	0.00125	5.29588	Up	7	12.79245	12.27743	12.31050	9.80832	10.54440	9.81304
NM_001000627	Olr701_predicted	0.00104	3.99558	Up	3	5.37576	5.48705	5.67957	3.90762	3.48223	3.15731
NM_053677	Chek2	0.00009	4.79475	Up	12	8.86950	8.84403	8.67135	6.52976	6.75512	6.31564
NM_001108298	Cdc6	0.00004	4.32074	Up	10	6.27822	6.10731	6.20886	4.13715	4.21775	3.90566
NM_001000045	Olr1564	0.00050	4.37034	Up	11	8.59920	8.19315	8.01413	6.13911	6.33668	5.94745
NM_133554	Slc17a1	0.00021	4.07956	Up	17	9.61966	9.26160	9.35299	7.23515	7.61283	7.30103
NM_001134526	RGD1305298	0.00028	3.80143	Up	6	12.70549	12.87161	12.56917	10.78576	11.02570	10.55518
NM_001044267	MGC112775	0.00171	4.73274	Up	5	11.89504	11.26799	11.38203	9.38440	9.60196	8.83066
NM_001105892	Dscr6	0.00213	4.35798	Up	11	13.28813	12.66876	12.81105	10.74262	11.23262	10.42171
NM_001000743	Olr110_predicted	0.00148	4.29979	Up	1	8.85370	8.41559	8.83855	6.45820	7.04732	6.28952
NM_001009177	Stfa3l1	0.00395	3.52549	Up	11	10.36907	9.74567	9.85851	8.14118	8.59783	7.78076
NM_022927	Mid1	0.00299	3.42529	Up	X	12.30728	11.74943	11.98512	10.15123	10.66069	9.90122
NM_198200	P518	0.00356	10.05736	Down	3	2.53407	3.09987	4.04598	7.14801	6.07090	6.45154
NM_019265	Scn11a	0.01023	6.57096	Down	8	2.53407	2.81928	2.83517	5.38697	6.48818	4.46168
NM_053744	Dlk1	0.00305	4.86921	Down	6	7.49082	8.48974	8.07381	10.28931	10.66765	9.94847
NM_173319	LOC286985	0.00018	3.94332	Down	1	2.53407	2.95543	2.97305	4.73203	4.86678	4.80198
NM_001106875	Acer1	0.02224	3.93238	Down	Un	3.39316	3.63306	2.98687	5.32956	4.41967	6.19006
NM_053822	S100a8	0.02769	4.93494	Down	2	4.79954	2.68874	2.88047	5.87408	5.83380	5.56997
NM_001033075	Defa7	0.00194	3.43660	Down	16	5.59667	4.78587	5.36146	6.97259	7.13337	6.98098
NM_001101018	Oaz3	0.02678	3.46768	Down	2	5.80303	4.00098	4.82002	6.60239	6.61141	6.79215
NM_031506	Cftr	0.01607	3.90136	Down	4	4.62705	3.08700	3.48477	5.67597	5.42079	5.99400
NM_001109547	Fam101a	0.00538	3.39621	Down	12	6.88143	5.85283	6.07142	8.08245	7.88576	8.12925

### Validation of the Microarray Data Using qRT-PCR

**Fig. 4:**
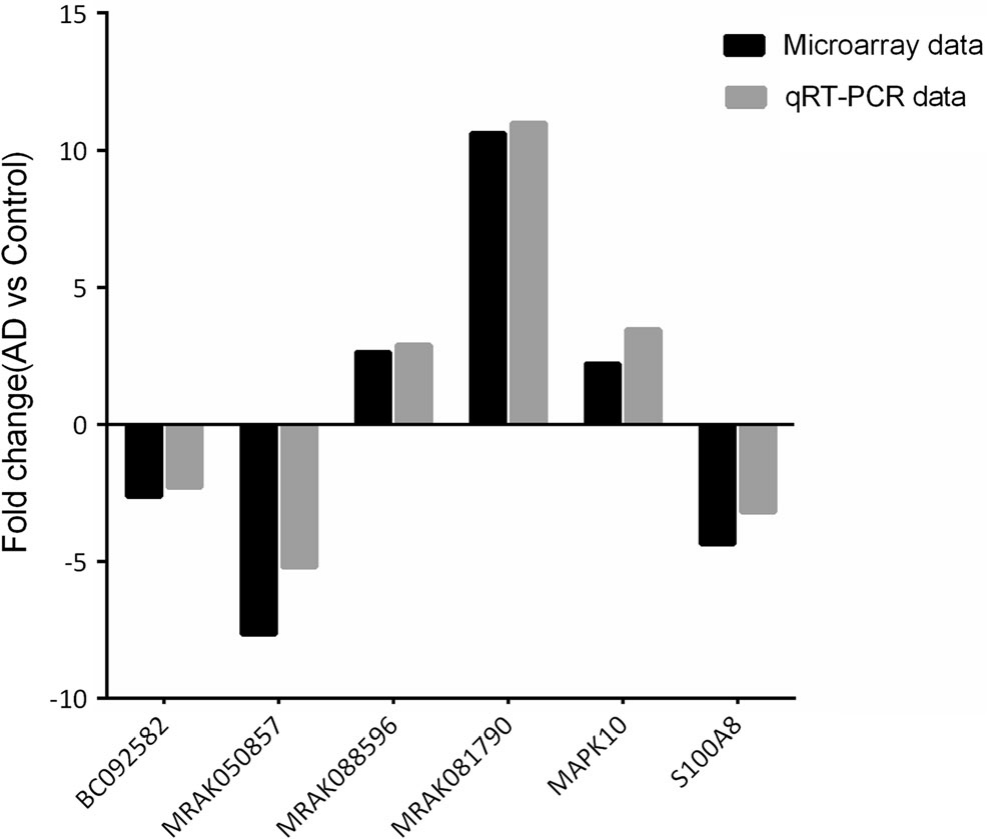
The differential expression of lncRNAs and mRNAs was validated by quantitative real-time PCR (qRT-PCR). The data showed that the expression levels of the lncRNAs BC092582 and MRAK050857 and the mRNA S100A8 were down-regulated and that the lncRNAs MRAK088596 and MRAK081790 and the mRNA MAPK10 were up-regulated in the hippocampal tissue samples from AD rats relative to the control rats. The heights of the columns in the chart represent FCs. The qRT-PCR results were consistent with the microarray data

A total of six dysregulated lncRNAs and mRNAs were randomly selected to validate the microarray results using qRT-PCR. Consistent with the microarray chip data, the qRT-PCR results demonstrated that the lncRNAs MRAK088596, MRAK081790 and MAPK10 were up-regulated and that BC092582, MRAK050857 and S100A8 were down-regulated in the AD rats compared with the controls (Fig. 4).

### Gene Enrichment and KEGG Pathway Analyses

To predict the functions of the lncRNAs, we employed a previously described method [27]. In brief, we first identified and then conducted a functional enrichment analysis of the mRNAs co-expressed with each of the differentially expressed lncRNAs. The enriched functional terms were used as the predicted functional terms for each given lncRNA.

**Fig. 5:**
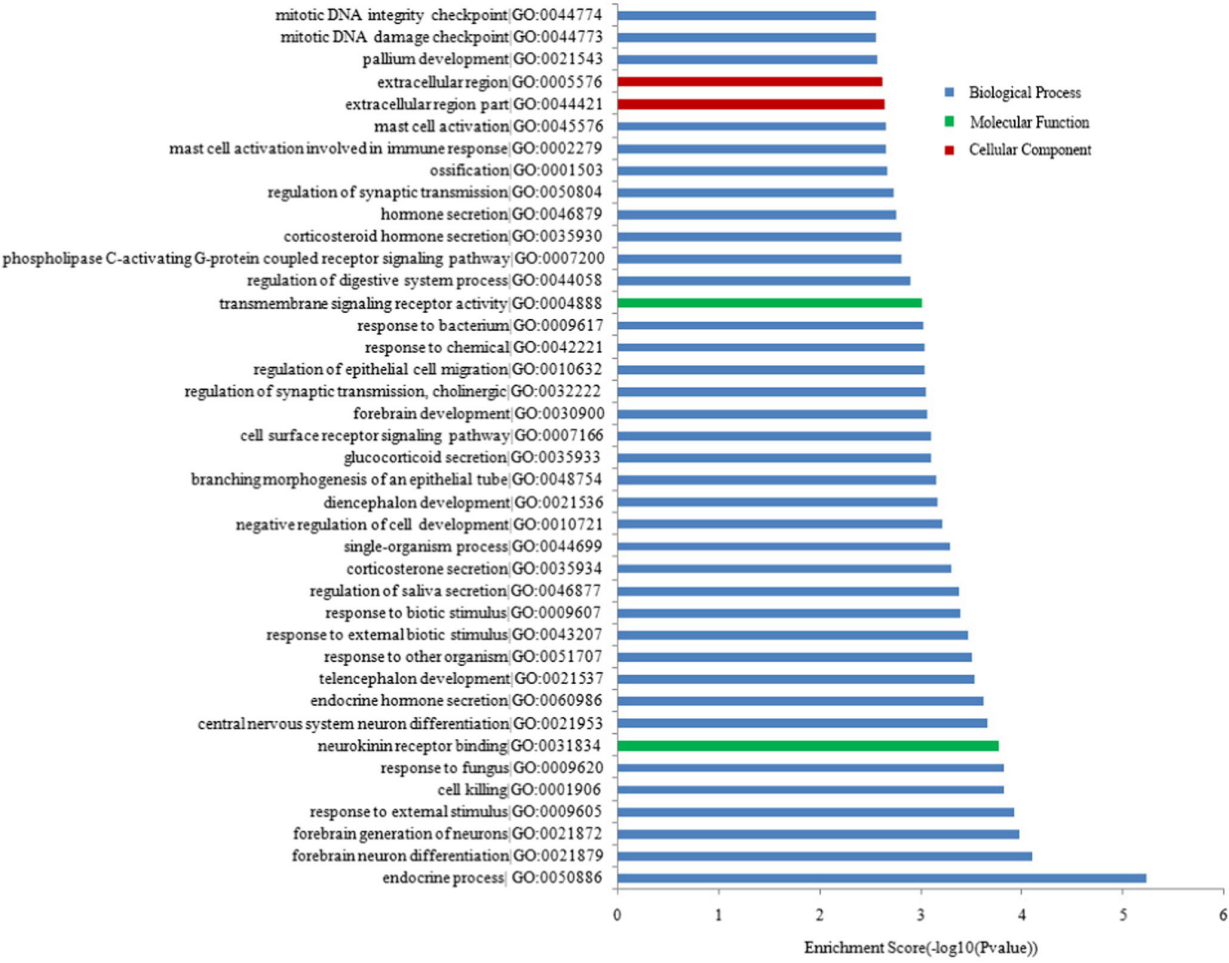
Top 40 GO terms for the differences in co-expressed lncRNA genes in AD animals and the controls. The GO enrichment analysis provided a controlled vocabulary to describe the co-expressed genes of the differentially expressed lncRNAs. The ontology covered three domains: biological process, cellular component, and molecular function

**Fig. 6:**
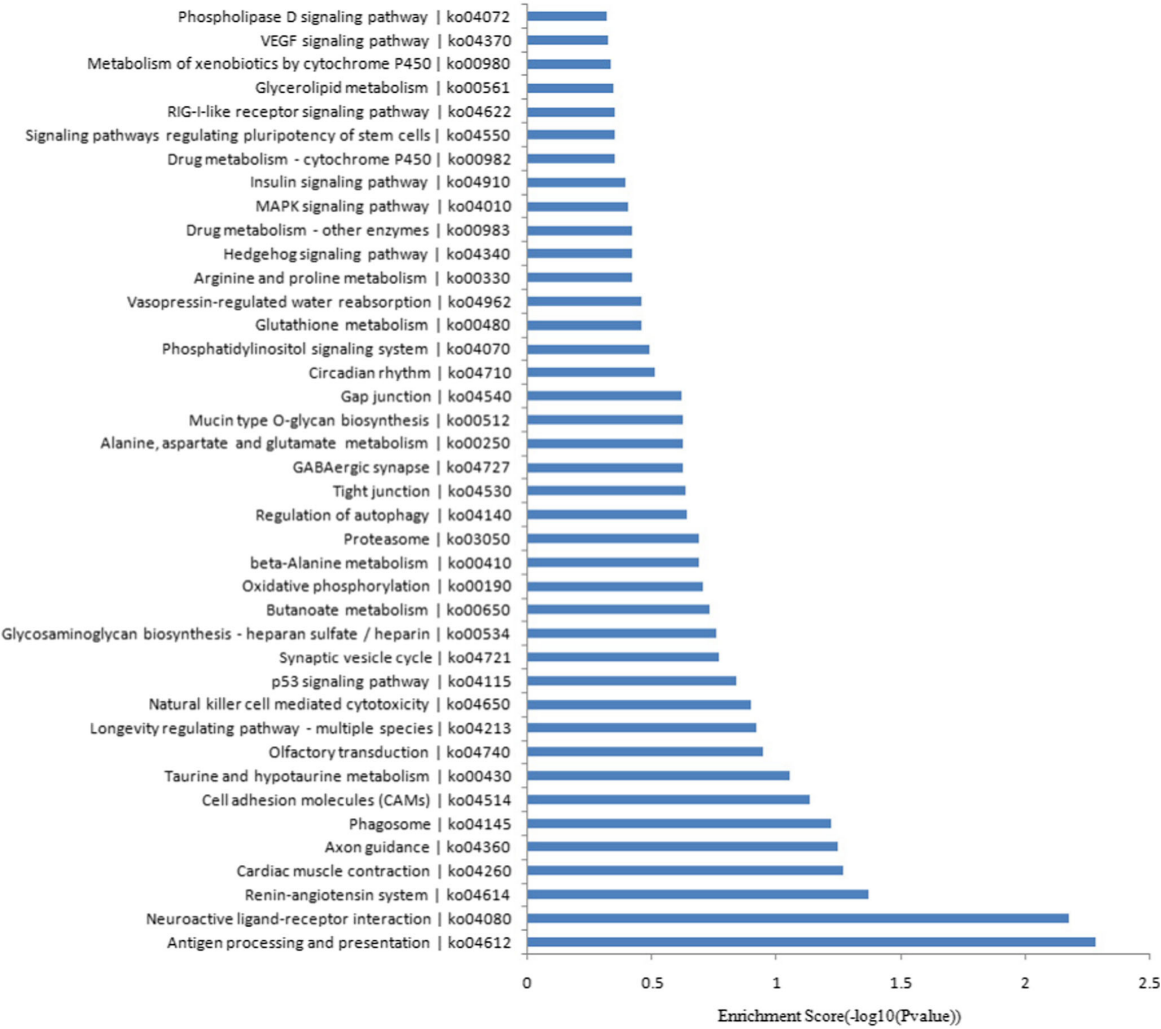
KEGG pathways analysis. Top 40 pathways for the differences in lncRNA genes co-expressed in AD animals and the controls

As shown in Fig. 5, the GO analysis indicated that the most enriched GO terms targeted by the mRNAs co-expressed with lncRNAs were endocrine process (ontology: biological process, GO:0050886), extracellular region part (ontology: cellular component, GO:0044421) and neurokinin receptor binding (ontology: molecular function, GO:0031834). Furthermore, the KEGG pathway analysis indicated that the mRNAs co-expressed with lncRNAs were involved in the regulation of antigen processing and presentation, neuroactive ligand-receptor interaction, axon guidance, and the synaptic vesicle cycle, among others. The top 40 KEGG pathways are listed in Fig. 6.

### lncRNA-mRNA Network Analysis

**Fig. 7:**
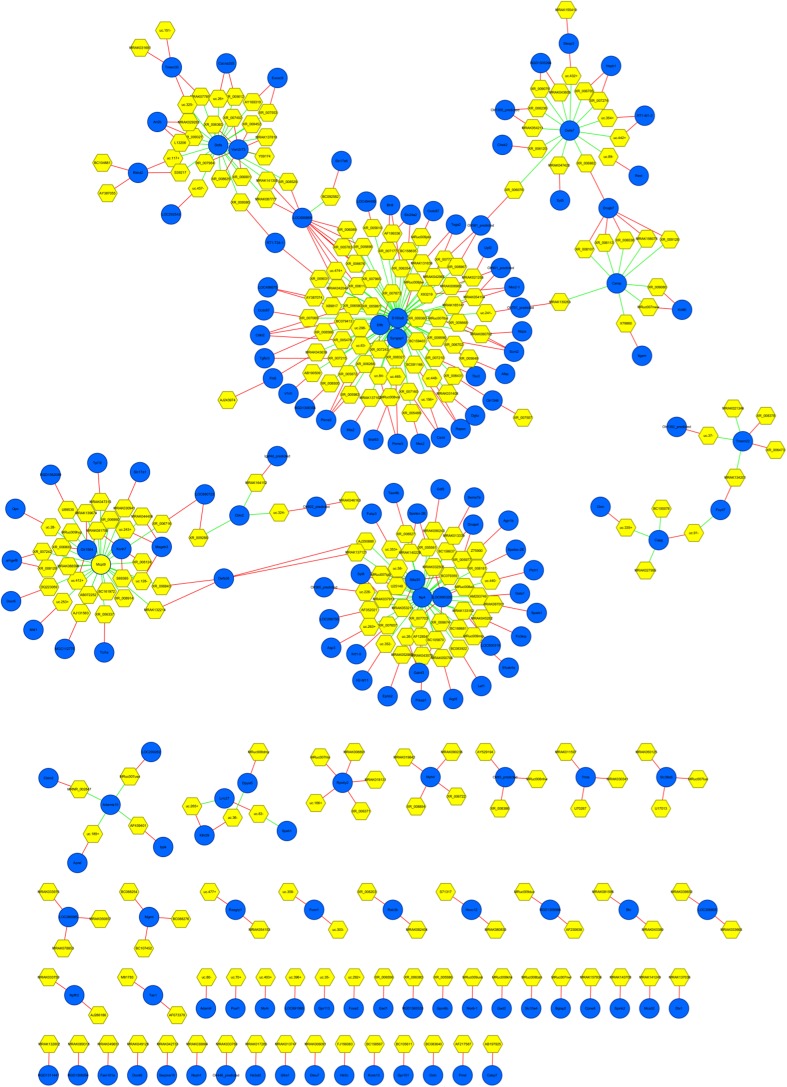
lncRNA-mRNA-network analysis. *Blue nodes* represent dysregulated lncRNAs, *yellow nodes* represent dysregulated mRNAs. The *red lines* between lncRNAs and mRNAs indicate a negative correlation, while the *green lines* indicate a positive correlation

As shown in Fig. 7, the whole co-expression network profile consisted of 454 network nodes and 478 connections among 168 differentially expressed mRNAs and 286 differentially expressed lncRNAs. There were 200 negative and 278 positive interactions within the network. Moreover, our data showed that one mRNA may correlate with 1–66 lncRNAs and that one lncRNA may correlate with 1–2 mRNAs.

## Discussion

Analysing the expression profiles of lncRNAs may provide new insights into our understanding of the aetiology and pathophysiology of AD. In previous studies, D.Y. Lee et al. examined the dysregulated expression of intergenic lncRNAs (lincRNAs), a significant subgroup of lncRNAs, in a triple transgenic model of AD (3xTg-AD) [16], X. Zhou et al. identified AD-associated lncRNAs based on post-mortem tissue samples of AD patients [14]. Additionally, *M. magistri* et al. identified several annotated and non-annotated lincRNAs that are differentially expressed in the hippocampus in late-onset Alzheimer’s disease (LOAD) [15]. Despite these previously reported findings, the roles of lncRNAs in AD remain largely unknown because none of the currently available models recapitulate all aspects of human AD [28].

As a useful experimental animal model of AD emphasizing the inflammatory component of the disease pathology, the Aβ_1–42_ infusion model strongly complements the use of transgenic animal models in advancing our understanding of AD [29]. However, comprehensive studies into the special expression patterns of lncRNAs in an Aβ_1–42_ infusion AD model have not been reported. Thus, to the best of our knowledge, this study is the first to describe hippocampal lncRNAs in an Aβ_1–42_ infusion AD model and further our understanding of lncRNAs that are associated with the pathogenesis of AD.

In the present study, we identified 315 lncRNAs and 311 mRNAs using a second-generation lncRNA microarray. Of these, 238 lncRNAs and 191 mRNAs were found to be significantly up-regulated in the AD rats compared with the control rats (FC ≥2.0, *p* < 0.05). In addition, several of the dysregulated lncRNAs and mRNAs, we identified were randomly chosen for qRT-PCR validation, and the results confirmed the microarray analysis findings to some extent. Among the dysregulated lncRNAs, 115 lincRNAs were found. Regarding the previous studies mentioned above, 472 significantly dysregulated mRNAs and 205 lincRNAs were identified in 3xTg-AD mice [16], 64 significantly dysregulated lincRNAs were found in AD patients [14], and 89 lincRNAs were found to be differentially expressed in the hippocampus of LOAD patients [15]. Taken together, these findings will likely lead towards a better understanding of the function of dysregulated lncRNAs in the neuropathogenesis of AD.

To predict the potential functions of the differentially expressed lncRNAs identified in this study, GO and KEGG pathway analyses were performed using the coding genes associated with the significantly differentially expressed lncRNAs. GO analysis revealed that these lncRNAs are involved in such biological processes as synaptic transmission regulation, cholinergic regulation, central nervous system neuron differentiation, external stimulus response processes and endocrine processes, all of which are important in learning and memory, as well as the development of AD. KEGG pathway analysis indicated that the genes associated with the dysregulated lncRNAs in the AD group are involved in the neuroactive ligand-receptor interaction, the renin-angiotensin system, axon guidance, and the PI3K-Akt, MAPK, and mTOR signalling pathways. Among these, the PI3K-Akt, MAPK, and mTOR signalling pathways play important roles in long-term learning and memory, such as in neurocyte nutrition, encoding protein synthesis regulation for memory formation in the hippocampus, and memory production and consolidation [30–32].

Furthermore, of these dysregulated lncRNAs, we identified AJ131563 and MRAK043570, which are associated with the insulin signalling pathway. Growing evidence suggests that the deregulation of insulin signalling in the brain plays an important role in the development of AD, which is involved in numerous molecular pathogeneses, including APP overexpression, Aβ accumulation, tau hyperphosphorylation, neuroinflammation, oxidative stress promotion and synaptic failure [33, 34]. On the other hand, the lncRNA MRAK043570 is significantly associated with the PI3K-Akt, mTOR, FoxO and AMPK signalling pathways, which are altered in brains with AD [35, 36]; in addition, all these pathways are linked to the regulation of autophagy, which is strongly regarded as one of the major pathogenic mechanisms of AD [37, 38]. These findings suggest that altered lncRNAs may be involved in AD-associated signalling pathways. However, our knowledge about the potential functions of these dysregulated lncRNAs in the neuropathogenesis of AD remains limited. Thus, further investigation is of great importance.

In the present study, we also employed an lncRNA-mRNA network analysis to identify interactions between differentially expressed mRNAs and differentially expressed lncRNAs, as previously described [39, 40]. The co-expression network reported here was constructed based on the 315 differentially expressed lncRNAs and the 311 differentially expressed mRNAs distinguishing the AD rats from the control rats. Our results showed that a total of 168 lncRNAs and 286 mRNAs were included in the co-expression network, which consisted of 454 network nodes and 478 connections. We also found that S100a8, an mRNA, was correlated with up to 66 lncRNAs; similarly, Np4 was correlated with 41 lncRNAs, Mcpt9 with 27 lncRNAs, Defa with 24 lncRNAs. Interestingly, a previous study has demonstrated that four mRNAs (S100a8, Np4, Mcpt9, and Defa) were involved in the neuroinflammatory responses in the frontal cortex of ageing female rats [41]. Because neuroinflammatory responses have been implicated as a significant contributor to AD pathogenesis [42], the specific roles of these mRNAs with respect to the neuropathology of AD deserve further investigation. In addition, S100a8 is a ligand of the receptor for advanced glycation end products, which is a receptor in the immunoglobulin superfamily that also binds other ligands, including advanced glycation end products, the high-mobility group protein B1 (HMGB1), and Aβ. In addition, S100A8 could modulate APP processing towards increased β-secretase activity and the production of long, more amyloidogenic, Aβ peptides [43].

We also found that 172 lncRNAs interacted with 5 mRNAs that are mainly involved in the neuroactive ligand-receptor interaction, lysosome and renin-angiotensin system signalling pathways, which have been demonstrated to play important roles in the pathophysiology of AD and could serve as potential therapeutic targets [44–46]. The co-expression network suggests that the inter-regulation of lncRNAs and mRNAs is involved in AD and warrants further study.

Although altered lncRNAs and mRNAs were identified and their possible roles in the pathophysiology of AD were investigated in this study, several limitations should be acknowledged. First, the analysis was only performed using the hippocampus of AD animals. Global lncRNA and mRNA changes in the blood and cerebral spinal fluid of the same AD model animals should be also determined in further studies to more accurately reflect the pathophysiology of AD. Second, the present study only predicted lncRNA functions through investigating the functional significance of the mRNAs co-expressed with the differentially expressed lncRNAs. Third, gene expression microarrays have a limited dynamic range and lack the ability to identify novel features, such as splice isoforms or fusion transcripts. RNA-seq technology allows the discovery of previously inaccessible complexities in the transcriptome, such as allele-specific expression and novel promoters and isoforms. However, the resulting datasets are large and complex, and their interpretation is not straightforward [47]. Fourth, similar to previous studies [47, 48], we also only performed the minimal number of experiments, which may result in an underestimation of the number of altered lncRNAs and mRNAs. Larger sample sizes could achieve more optimal results. Additionally, further research should select from 8 to 10 up-regulated and 8 to 10 down-regulated lncRNAs for microarray validation and include a larger sample size than that in the present study. Future studies that overcome the limitations mentioned above are merited.

In conclusion, the present study uses microarray data to reveal for the first time that the hippocampal expression patterns of lncRNAs are significantly altered in AD. In addition, the data indicate that aberrantly expressed lncRNAs participate in several specific biological processes and are involved in related pathways that may contribute to the pathogenesis of AD. While these findings provide newfound information regarding the potential role of lncRNAs in AD, further research is required to fully elucidate the detailed molecular mechanisms underlying the action of significantly dysregulated lncRNAs.
